# Improving Engagement With Breast Cancer Screening Through Structured Telephone Outreach Among Uninsured Women

**DOI:** 10.7759/cureus.116273

**Published:** 2026-09-15

**Authors:** Bradley W Cantley, Alex Morrison, Kevin Neubrecht, Trent Agee, Reece George, Chandler Pugh, Cleon Rogers

**Affiliations:** 1 Physician Assistant Studies, Samford University, Homewood, USA; 2 Medical School, Edward Via College of Osteopathic Medicine, Auburn, USA; 3 Internal Medicine, Christ Health Center, Birmingham, USA

**Keywords:** breast cancer screening, health disparities, mammography, preventive medicine, primary care, telephone outreach, uninsured populations

## Abstract

Background

Financial barriers, limited healthcare engagement, and gaps in preventive care contribute to delayed breast cancer screening among uninsured women. This study evaluated mammography completion within 60 days and secondary measures of breast cancer screening engagement following structured telephone outreach combined with facilitated scheduling through a free state-sponsored screening program among uninsured women overdue for mammography.

Hypothesis

We hypothesized that structured telephone outreach offering facilitated access to free mammography would support patient engagement across the screening cascade, including successful contact, referral acceptance, and facilitated scheduling, with mammography completion assessed as the primary clinical outcome.

Methods

This single-arm quality improvement study was conducted at a federally qualified health center in Birmingham, Alabama, United States. Eligible participants were uninsured women aged 52-74 with no documented mammogram within the preceding 24 months. Electronic medical record (EMR) review was used to verify screening status and identify potential barriers to screening. Trained medical students conducted standardized telephone outreach that included notification of overdue screening status, brief education regarding early detection, and an offer to schedule a free mammogram through the Alabama Breast and Cervical Cancer Early Detection Program (ABCCEDP). Up to three call attempts were made within 48 hours. The primary outcome was documented mammography completion within 60 days of initial telephone outreach. Secondary outcomes included mammography completion within 30 days, successful patient contact, referral acceptance, completion of an ABCCEDP visit, mammography scheduling, and documented barriers to screening.

Results

Among 361 eligible patients, 110 (30.5%) were successfully contacted, of whom 70 (63.6%) accepted referral and were transferred to scheduling. Among the 70 referred patients, two (2.9%) had documented mammography completion within 30 days and three (4.3%) within 60 days. At 60 days, three patients (4.3%) had completed an ABCCEDP visit only, 12 (17.1%) had completed an ABCCEDP visit and scheduled a mammogram, and 52 (74.3%) had no documented follow-through. Common patient-reported barriers included fear of abnormal results, competing health priorities, transportation or time limitations, preference for discussing screening with a primary care provider, and personal preference.

Conclusions

Structured telephone outreach paired with facilitated scheduling appears to be a feasible, low-cost strategy to support engagement with breast cancer screening, including patient contact, referral acceptance, and facilitated scheduling, among uninsured women. However, persistent barriers beyond the initial referral process highlight the need for enhanced patient navigation and continued follow-up to improve screening completion.

## Introduction

Breast cancer remains a leading cause of cancer-related morbidity and mortality among women in the United States and within the state of Alabama. In Alabama, the female breast cancer mortality rate (21.3 per 100,000) exceeds the overall United States rate (20.2 per 100,000). Substantial racial disparities persist, with African American women experiencing markedly higher mortality than White women (27.4 vs. 19.4 per 100,000). Although Alabama’s overall breast cancer incidence (121.8 per 100,000) is slightly lower than the national rate (126.8 per 100,000), African American women in the state have a higher incidence than White women (126.8 vs. 119.4 per 100,000) [1]. These data highlight the continued need for targeted interventions to improve early detection and reduce disparities in breast cancer outcomes.

Breast cancer risk is influenced by both nonmodifiable and modifiable factors [2]. Static risk factors include increasing age, pathogenic variants in *BRCA1* or *BRCA2*, early menarche, late menopause, dense breast tissue, prior personal or family history of breast or ovarian cancer, and prior chest radiation. Modifiable contributors include obesity, physical inactivity, alcohol and tobacco use, nulliparity, lack of breastfeeding, and use of hormone replacement therapy (ADPH) [2]. Because many women at risk remain asymptomatic until later-stage disease, adherence to evidence-based screening recommendations is critical for early detection and mortality reduction.

Multiple professional organizations provide guidance on breast cancer screening. The United States Preventive Services Task Force recommends biennial mammography for women aged 40-74 years (Grade B recommendation) [3]. The American Cancer Society recommends optional annual screening beginning at ages 40-44, annual screening from 45-54, and transition to biennial or continued annual screening at age 55 and older while life expectancy remains at least 10 years; women at high risk should begin annual MRI and mammography at age 30 [4]. Similarly, the American College of Obstetricians and Gynecologists advises offering screening starting at age 40 using shared decision-making and initiating no later than age 50, with mammography every one to two years through at least age 75 [5]. Despite these consistent recommendations, adherence remains suboptimal. In 2022, mammography prevalence within the last two years among women aged 40 and older was 69% in Alabama compared to a national prevalence of 67% [6].

Persistent gaps in breast cancer screening adherence are closely linked to deficits in knowledge, financial burden, and healthcare engagement through patient-provider relationships. Among women considered at elevated risk, limited understanding of personal screening eligibility, individual risk status, and available screening modalities represents a central barrier to participation. Many women remain unaware of imaging options beyond standard mammography, and substantial uncertainty exists regarding the purpose, timing, and benefits of recommended screening examinations. In one qualitative study, 67% of high-risk participants who had not received a breast MRI reported that they were unaware of screening modalities beyond mammography [7]. In addition to lack of knowledge, patients often delay or refuse screening due to fear of bad results and negative outcomes. A study with over 5,500 participants conducted in Alabama demonstrated that fear of bad results led to the longest delay in screening time completion [8]. These findings underscore the importance of targeted educational outreach to improve informed decision-making and timely screening completion. Broader knowledge gaps across racial, ethnic, and rural populations further reinforce this need.

Financial burden further constrains screening participation and is one of the most frequently reported structural barriers. Concerns about lack of insurance coverage, anticipated out-of-pocket costs, and the potential financial consequences of a cancer diagnosis may deter women from obtaining recommended screening, even when preventive services are available [7]. Lack of medical insurance has been identified as a primary barrier to adherence and follow-up in cancer screening programs [9]. Furthermore, a study performed in South Carolina demonstrated that uninsured women were associated with higher stages of breast cancer at diagnosis, and insured women experienced significantly increased survival odds 3.28 compared to these uninsured women [10]. Misconceptions regarding insurance coverage for screening services and downstream care compound these concerns, particularly for supplemental imaging such as MRI [7]. Socioeconomic status and insurance access therefore remain critical determinants of screening utilization.

The strength of the patient-provider relationship also substantially influences screening behavior. Direct recommendations from a trusted healthcare professional consistently increase the likelihood of screening completion, especially when accompanied by clear communication about individual risk and expected benefits [7]. Positive therapeutic relationships and provider engagement thus function as key facilitators capable of mitigating both informational and motivational barriers to preventive care.

Despite these challenges, mammography remains the cornerstone of early breast cancer detection. Digital mammography demonstrates high specificity (99%) and moderate sensitivity (78%) in average-risk populations, with reduced performance in women with dense breast tissue but improved detection through advances such as tomosynthesis and three-dimensional (3D) imaging [2,11]. These technological improvements enhance identification of small, clinically occult tumors while reducing false-positive findings, supporting continued reliance on mammography within population-level screening strategies.

Evidence-based public health interventions can meaningfully increase screening uptake. A key example of this is the Alabama Breast and Cervical Cancer Early Detection Program (ABCCEDP), which attempts to address financial barriers to routine mammography completion through offering free breast cancer screening to low-income, uninsured patients in the state of Alabama [1]. While little research has been published regarding the efficacy of this program, a similar program on a national scale, the National Breast and Cervical Cancer Early Detection Program (NBCCEDP), has been shown to increase the number of lifetime mammograms in uninsured patients [12]. Educational initiatives, individualized reminders, reduction of out-of-pocket costs, and provider assessment with feedback are recommended by the Community Preventive Services Task Force as effective approaches to improve breast cancer screening rates [13]. Additional research demonstrates that structured outreach interventions, including reminders and community-based engagement strategies, can further improve mammography completion, particularly in underserved populations [9].

Together, these findings highlight the need for targeted, low-cost interventions that directly address knowledge deficits, financial constraints, and limited healthcare engagement. Programs that combine cost-free screening opportunities with proactive patient outreach may be especially useful for uninsured women who encounter the greatest structural barriers to preventive services.

Although telephone outreach has been used to promote breast cancer screening, less is known about the feasibility and screening outcomes associated with combining direct telephone notification of free mammography with facilitated access to screening through programs such as the ABCCEDP among uninsured women. In particular, it remains unclear whether reducing both informational and access-related barriers through structured outreach translates into screening engagement and completion, or what barriers persist despite these efforts. Evaluating this approach may provide actionable evidence to guide strategies aimed at improving breast cancer screening engagement and early detection among uninsured women. Therefore, the primary objective of this quality improvement study was to evaluate documented mammography completion within 60 days following structured telephone outreach and facilitated access to free screening among uninsured women overdue for mammography. Secondary objectives were to evaluate 30-day mammography completion, successful patient contact, referral acceptance, completion of an ABCCEDP visit, mammography scheduling, and barriers encountered across the screening cascade.

## Materials and methods

This single-arm quality improvement study evaluated mammography engagement and completion following structured telephone outreach among women overdue for breast cancer screening. The study was conducted at Christ Health Center, a federally qualified health center in Birmingham, Alabama, United States, from January 2026 until May 2026. The study protocol was approved by the Samford University Institutional Review Board (application number: EXMT-HP-26-SUM-2) and was deemed exempt from subject informed consent.

Participants

The study population consisted of uninsured women aged 52-74 years receiving primary care at Christ Health Center who were overdue for screening mammography, defined as no documented mammogram within the previous 24 months in the electronic medical record (EMR). These criteria were based on Athena EMR quality measures [14] corresponding to the 2016 United States Preventive Services Task Force (USPSTF) recommendations [15] and Healthcare Effectiveness Data and Information Set (HEDIS) measures for biennial breast cancer screening among women aged 50-74 years [16]. The lower age threshold of 52 years was used to identify women who were at least 24 months beyond the initial screening eligibility age of 50 years without a documented mammogram.

A structured EMR review was performed for all eligible patients to verify screening status and identify documentation gaps. Patients with evidence of completed screening that was not captured in structured EMR fields were excluded from outreach when appropriate. For the remaining patients, EMR-documented factors contributing to screening noncompletion, including absent referrals, prior nonadherence, and patient refusal, were recorded. Screening status was based on available EMR documentation, including documentation of mammography performed outside the health system when available.

Telephone outreach

Trained medical students conducted standardized telephone outreach using a structured script. Up to three telephone call attempts were made for each patient within a 48-hour period at varying times of the day. Before each outreach attempt, callers reviewed the patient's EMR to confirm overdue screening status and identify previously documented barriers to screening. A voicemail was left for every failed attempt to contact the patient. For patients with a preferred language other than English, a telephone-based interpreter service was utilized. The interpreter was instructed to identify themselves and the caller before proceeding with the standardized script.

During successful telephone encounters, callers first obtained verbal consent to discuss breast cancer screening. Patients were informed that they were overdue for screening mammography and received brief education emphasizing the importance of early detection. Patients were then offered facilitated scheduling for a free screening mammogram through the ABCCEDP. Those who agreed were immediately transferred to scheduling services to arrange their appointment. For patients who declined screening, callers documented the primary reason for nonparticipation. Following each call, standardized documentation was completed to record patient reachability, referral acceptance, and any EMR-documented or patient-reported barriers to screening.

Outcomes and measures

The primary outcome was documented mammography completion within 60 days of the initial telephone outreach attempt. Mammography completion was defined as documentation confirming that the screening mammogram was performed.

Secondary outcomes included mammography completion within 30 days; successful patient contact, defined as direct telephone contact with the patient; referral acceptance, defined as patient agreement to proceed with facilitated scheduling for a screening mammogram through the ABCCEDP; documented completion of an ABCCEDP visit; and mammography scheduling, defined as documentation of a scheduled mammography appointment. These measures represented sequential stages of the screening cascade but were analyzed as distinct outcomes rather than as a single composite progression endpoint.

Additional secondary outcomes included EMR-documented barriers to screening, including absent referrals, incomplete follow-through, and documentation gaps, as well as patient-reported barriers, including fear of diagnosis, competing medical concerns, and personal preference.

Mammography referral acceptance and initial scheduling at outside facilities were documented based on patient report when applicable. Final verification of mammography appointment scheduling and mammography completion occurred after Christ Health obtained records from the imaging facility.

Data management

All study data were recorded in a secure, password-protected spreadsheet accessible only to authorized study personnel. Patients were identified solely by unique EMR identification numbers, and no protected health information was recorded. Data collected included EMR-documented reasons for screening noncompletion, telephone outreach outcomes, and patient-reported barriers among those who declined screening. Data integrity was maintained through periodic review for completeness and accuracy. In accordance with institutional policy, study data will be destroyed three years after study closure.

Statistical analysis

Data were collected and organized in Google Sheets (Google LLC, Mountain View, California, United States) and analyzed using descriptive statistical methods. Categorical variables were summarized as frequencies and percentages. Differences in outreach outcomes across age groups were evaluated using chi-square or Fisher's exact tests, as appropriate. Two-sided p-values <0.05 were considered statistically significant. Results were presented using bar graphs to illustrate outreach outcomes across age categories.

## Results

A total of 361 eligible uninsured patients overdue for breast cancer screening were identified during the study period. A total of 110 of 361 eligible patients (30.5%) were successfully contacted, while 251 (69.5%) could not be reached despite multiple outreach attempts. Among the 110 successfully contacted patients, 70 (63.6%) accepted referral and were transferred to scheduling, representing 19.4% of the overall eligible population. The remaining 40 contacted patients (36.4%) declined referral, representing 11.1% of the overall eligible population (Table 1).

**Table 1:** Summary of telephone outreach outcomes

Telephone Outreach Outcome	Frequency (Percentage)
Eligible patients identified	361 (100.0)
Successfully contacted	110 (30.5)
Unable to contact	251 (69.5)
Among successfully contacted patients (n = 110)	
Accepted referral for free mammography	70 (63.6)
Declined referral	40 (36.4)

**Figure 1:**
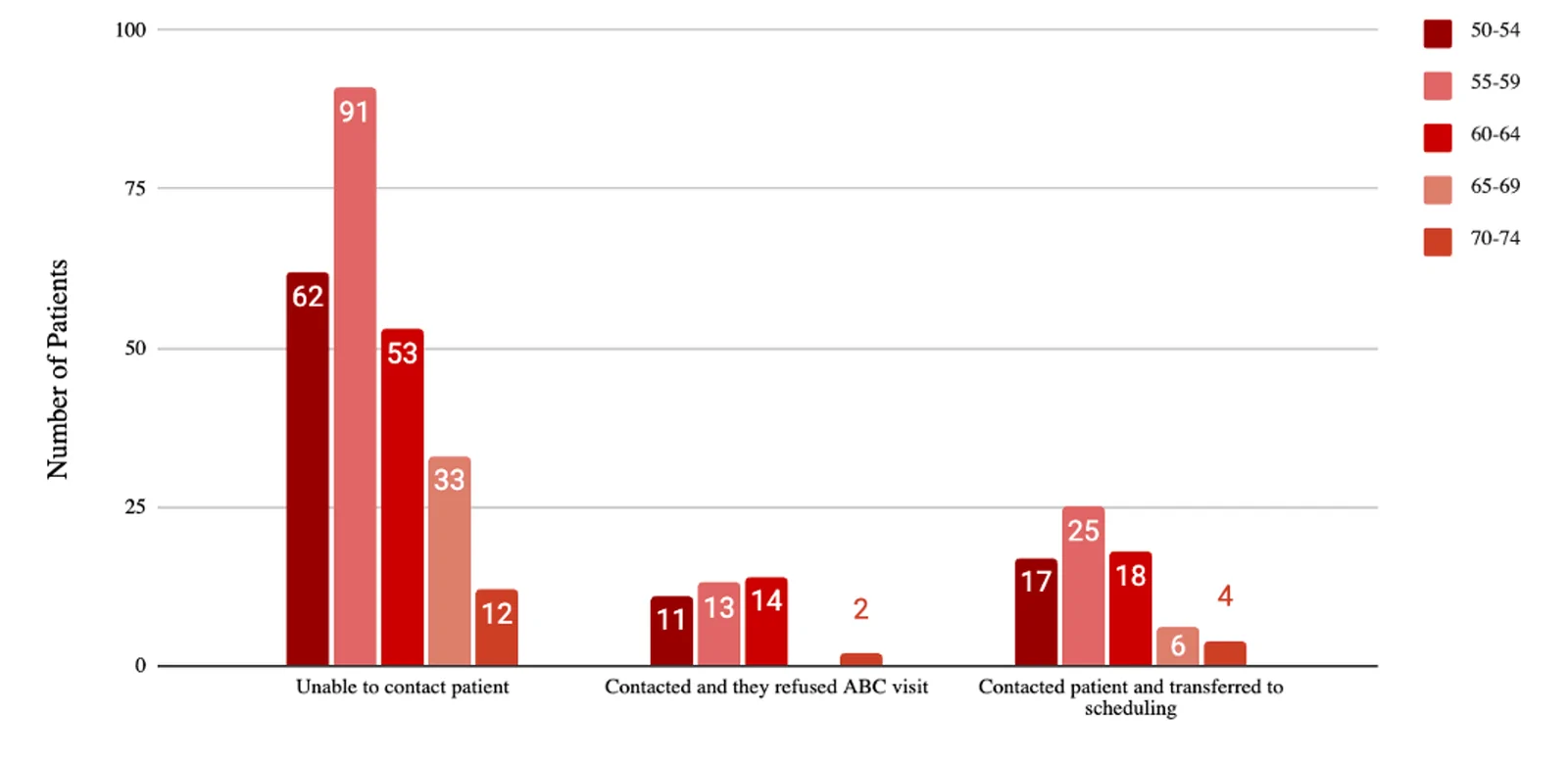
Outreach outcomes by age group This bar graph displays telephone outreach outcomes stratified by age group, including inability to contact, refusal of screening, and successful transfer to scheduling. ABC: Alabama Breast and Cervical Cancer Early Detection Program

The distribution of successfully contacted patients across age groups was relatively similar, with slightly higher numbers among patients aged 60-64 and 70-74 years. Referral acceptance was observed across all age groups, with the highest number of scheduling transfers occurring among patients aged 55-59 years (Figure 1). Referral acceptance did not significantly differ across age groups (χ² = 4.39, p = 0.356), though slightly higher in the 60-64 cohort. 

Of the 361 eligible patients, 156 (43.2%) had a preferred language other than English documented in the EMR. Of these, 151 (96.8%) were Spanish-speaking, with the remaining patients speaking Mongolian, Japanese, French, or Arabic. Among the 156 patients with a preferred language other than English, 30 (19.2%) were successfully contacted. Of those contacted, 24 (80.0%) accepted referral and agreed to be transferred to the scheduling team, while six (20.0%) declined screening. Among the 205 English-speaking patients, 80 (39.0%) were successfully contacted. Of those contacted, 46 (57.5%) accepted referral and agreed to be transferred to scheduling, while 34 (42.5%) declined screening. Thus, successful contact was lower among patients with a preferred language other than English compared with English-speaking patients (19.2% vs. 39.0%); however, among those successfully contacted, referral acceptance was higher among patients with a preferred language other than English (80.0% vs. 57.5%).

**Figure 2:**
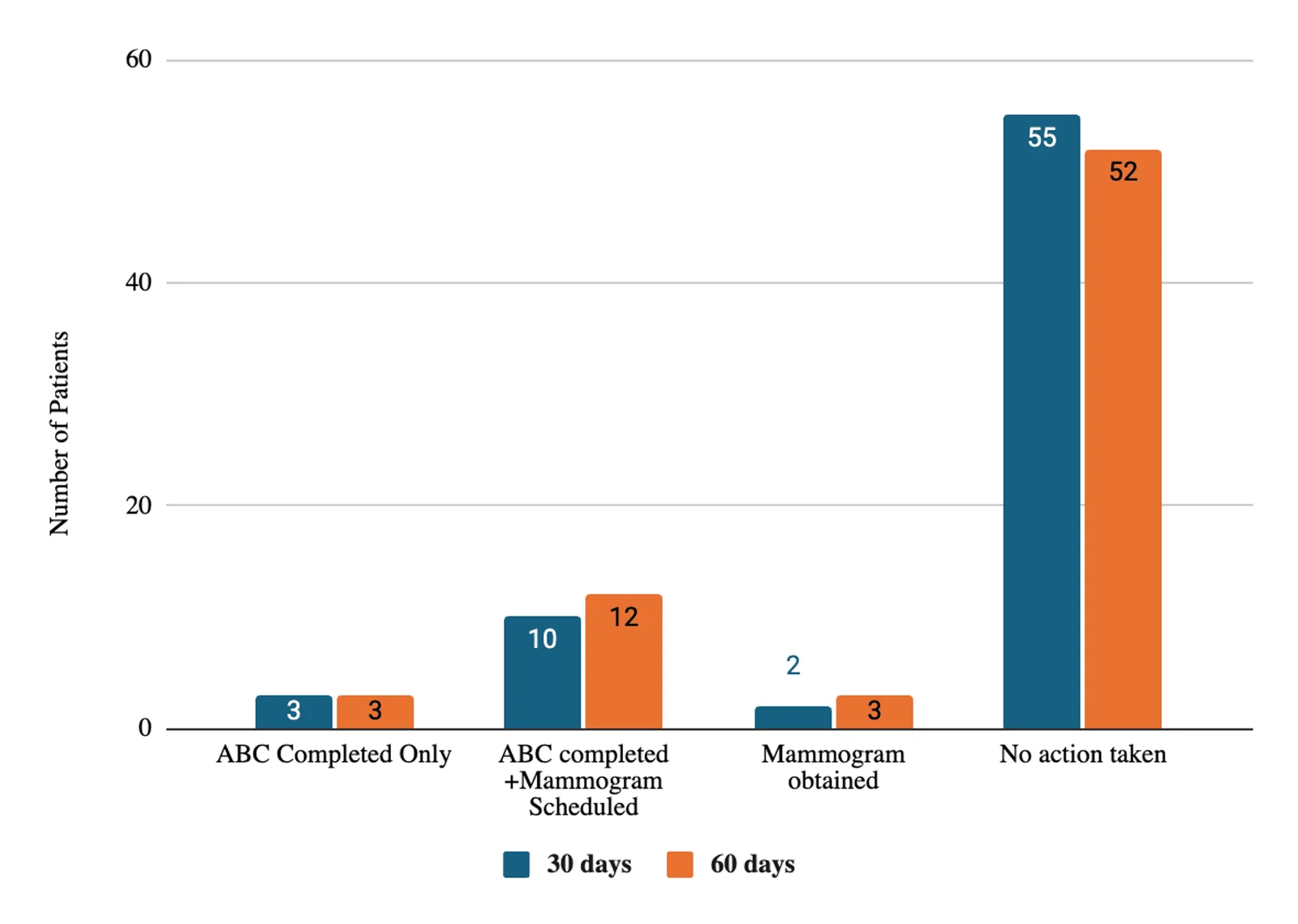
Intervention assessment at 30-day and 60-day time points This bar graph illustrates the outcomes of telephone outreach at 30- and 60-day intervals among patients who were successfully contacted and referred to the scheduling office. ABC: Alabama Breast and Cervical Cancer Early Detection Program visit

Detailed assessment of the primary and secondary follow-up outcomes was conducted among the 70 patients who accepted referral and were transferred to scheduling. At 30 days, three patients (4.3%) had completed only an ABCCEDP visit, 10 (14.3%) had completed an ABCCEDP visit and scheduled a mammogram, two (2.9%) had obtained a mammogram, and 55 (78.6%) had no documented follow-through. At 60 days, three patients (4.3%) remained categorized as ABCCEDP visit only, 12 (17.1%) had completed an ABCCEDP visit and scheduled a mammogram, three (4.3%) had obtained a mammogram, and 52 (74.3%) had no documented follow-through (Figure 2).

Additional secondary outcomes included EMR-documented and patient-reported barriers to screening. EMR review identified several contributors to delayed screening, including absence of referral placement, documented patient nonadherence, patient refusal, and lack of documented explanation. Patients who declined participation after contact commonly reported barriers such as fear of bad results, competing health priorities, lack of time or transportation, preference for discussing screening with a primary care provider, and personal preference.

## Discussion

This quality improvement initiative evaluated mammography completion and screening engagement following structured telephone outreach with facilitated access to free breast screening services among uninsured women overdue for mammography. The primary outcome, documented mammography completion within 60 days, occurred in three (4.3%) of the 70 patients who accepted referral and were transferred to scheduling. In contrast, secondary process outcomes demonstrated greater initial engagement, with 110 of 361 eligible patients (30.5%) successfully contacted and 70 of those contacted (63.6%) accepting referral and agreeing to transfer to scheduling. These findings demonstrate a distinction between successful initial engagement and completion of the clinical screening endpoint. Although structured outreach was effective in engaging a subset of patients in the initial stages of the screening cascade, these process outcomes did not consistently translate into mammography completion during the 60-day follow-up period. These findings are consistent with prior literature supporting structured outreach, reminders, and other targeted interventions as strategies to improve engagement with breast cancer screening services [13].

Despite successful referral acceptance among contacted patients, relatively few patients progressed through subsequent scheduling steps within the 30- and 60-day follow-up periods. This finding suggests that barriers persist even after initial patient engagement and highlights the need for continued navigation support beyond the initial outreach encounter. Additional strategies may be necessary to support patients through subsequent steps in the screening process. Patient navigation, reminder systems, transportation assistance, and longitudinal follow-up may help address barriers that persist after initial referral acceptance and support progression from scheduling to completed mammography. Integrating these strategies with structured telephone outreach may provide a more comprehensive approach to supporting patients throughout the screening cascade. Future studies may compare these findings with those of an insured cohort of women meeting similar age and breast cancer screening criteria who are not eligible for the ABCCEDP program.

EMR review further highlighted both patient and system-level contributors to missed screening opportunities, including patient nonadherence, absent referral placement, incomplete follow-through, and inconsistent documentation regarding overdue status. Patient-reported barriers such as fear of abnormal results, competing health priorities, and preference to discuss screening with a primary care provider further emphasize the importance of patient education, supportive communication, and provider engagement in improving screening adherence.

This study has several limitations. A substantial proportion of patients could not be reached, which may introduce selection bias, as individuals who are reachable may be more likely to schedule and complete mammography than those who are not reachable. Additionally, the single-arm design lacked a control or pre-intervention comparison group, limiting the ability to determine whether observed screening outcomes were attributable to the telephone outreach intervention. Furthermore, screening status was determined from available EMR documentation, and mammograms completed outside the health system may not have been captured, potentially resulting in misclassification of some women as overdue for screening. The study was conducted at a single federally qualified health center, which may limit generalizability to other populations. The relatively short follow-up period may also not capture delayed screening completion, as many local health care entities with the infrastructure to perform mammography currently have long wait times. Upon further review of outreach barriers, it was noted that the telephone interpreter service used for outreach displayed a Washington state phone number rather than a local area code corresponding to the study population. This discrepancy may have contributed to lower initial response rates, as unfamiliar out-of-state numbers may be perceived as telemarketing or spam calls. The broader political climate surrounding immigration policy may have also contributed to hesitancy among non-English-speaking patients to answer unfamiliar phone calls.

## Conclusions

Overall, structured telephone outreach combined with facilitated scheduling demonstrated measurable initial engagement with breast cancer screening among uninsured and underserved patients; however, documented mammography completion within 60 days remained low. Among patients successfully contacted, referral acceptance and facilitated scheduling demonstrated that the intervention was feasible for promoting early steps in the screening cascade, but these process outcomes did not consistently translate into completed mammography. Persistent barriers beyond initial patient engagement highlight the need for continued navigation and support to facilitate progression from referral to completed screening.

Ongoing efforts at Christ Health Center, including development of an on-site mammography suite, may further reduce barriers related to transportation and access and support continuity from outreach and referral to screening completion. Future studies incorporating a comparison group and longer follow-up are needed to determine whether structured telephone outreach and enhanced patient navigation result in improved mammography completion.
